# Computational Experiments Probing the Adaptability of the [NCCH_2_]^−^ Electronic Structure to Various Bonding Environments

**DOI:** 10.1002/cphc.202500580

**Published:** 2026-01-25

**Authors:** Jordan Rio, Jean‐François Brière, Hélène Gérard

**Affiliations:** ^1^ Sorbonne Université CNRS Laboratoire de Chimie Théorique LCT F‐75005 Paris France; ^2^ CNRS INSA Rouen Normandie Univ Rouen Normandie Univ Caen Normandie ENSICAEN Institut CARMeN UMR 6064 F‐76000 Rouen France

**Keywords:** electronic structures, ligand effects, metalation, molecular modeling, solvent effects

## Abstract

Using combined geometry optimization and electronic analyses, it is examined how metal nature (alkali and Cu(I)), solvation (THF), ligands, and aggregation modulate the N‐ versus C‐bonding balance in metalated acetonitrile. C‐binding is energetically favored in covalent Cu(I) complexes, while lithiated species prefer N‐binding. Surprisingly, N‐metalated species do not all exhibit the expected ketenimine‐like character (C=C=N, lone pair on N), but a nitrile‐like one (C^*b*^—C≡N, lone pair on C^*b*^) also emerges from the natural bond orbital analyses. Ketenimines are stabilized by polarizing or covalent M—N bonds, while nitriles are obtained with weakly coordinating cations or in anionic species. Notably, an external electric field can induce a similar electronic reorganization, thus revealing the electronic flexibility of metalated nitriles.

## Graphical abstract

Metalated nitriles are organometallic chameleons in solution, existing as a structural duality of N‐ and C‐metalated species exhibiting specific reactivities. With density functional theory (DFT) calculations and natural bond orbital analyses, it is explored how the metal nature (Li, Na, K, Cs, Cu(I)) and the chemical environment (ligand, solvation) impact this continuum. A divergence between metalation site and formal charge localization is evidenced.

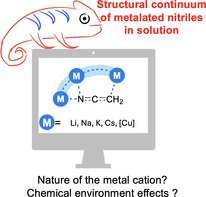

## 1 Introduction

Metalated nitriles are versatile synthetic tools for organic chemists. An important use of metalated nitriles is in cyanoalkylation and cyanoarylation reactions.^[^ ^1^, ^2^, ^3^, ^4^, ^5^, ^6^, ^7^, ^8^ ^]^ In these reactions, the metalated nitriles are typically generated in situ by deprotonation, halogen– or sulfoxide–metal exchange reactions.^[^ ^9^ ^]^ While these preparation methods are well established, the nature of the metal plays a critical role in dictating the electronic properties of the resulting metalated species, and the reactivity derived thereof, as testified by an insightful review on this topic.^[^ ^9^ ^]^

**Scheme 1:**
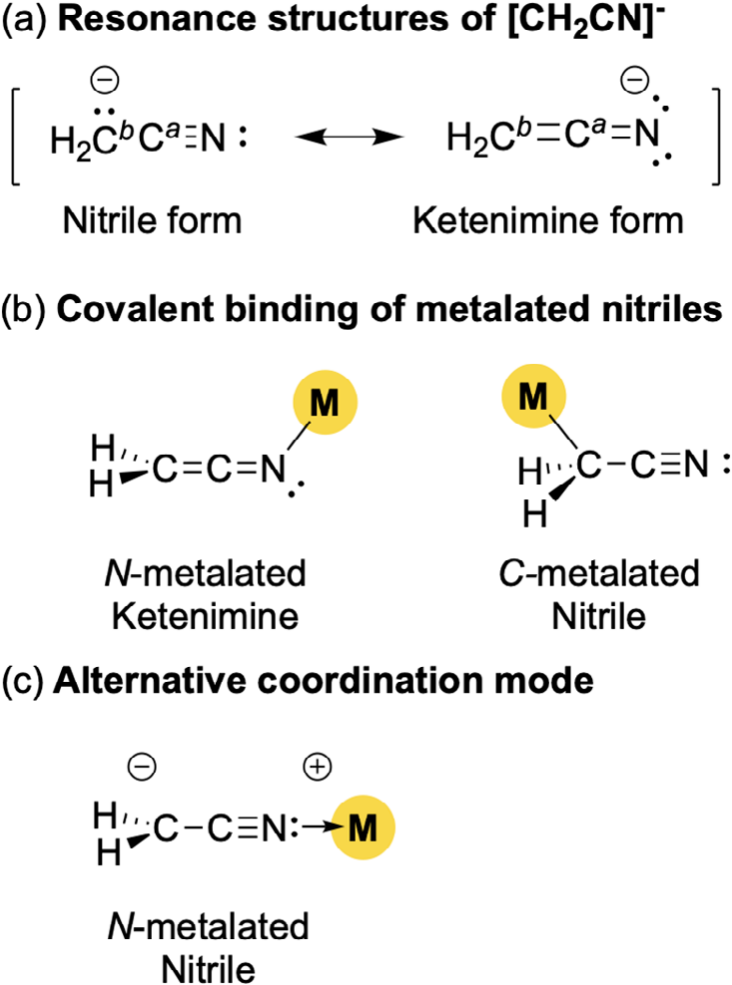
a) Resonance structures of [CH_2_CN]^−^ and b,c) structural continuum of metalated nitriles. M = alkali‐metal or transition‐metal catalyst.

In the absence of metal coordination, the acetonitrile anion [NCCH_2_]^−^, obtained by deprotonation of acetonitrile, predominantly exhibits a negative charge localized at the carbon atom adjacent to the cyanide functional group (C^*b*^ in **Scheme** **1**a) rather than at the nitrogen N atom of the ketenimine form.^[^ ^10^ ^]^ Ab initio calculations and QM/MM simulations of cyanoalkane deprotonation in water suggest that acetonitrile has a p*K*_a_ of 28.9, with its anionic form being stabilized primarily through inductive effect rather than by the mesomeric withdrawal capability of the cyanide functional group. In principle, the resonance forms of [NCCH_2_]^−^ allow for two possible metalated structures, namely, the N‐metalated ketenimine and C^*b*^‐metalated nitrile derivatives (Scheme 1b). However, this simplistic representation is incomplete, considering that some metalated species are indeed best described as N‐metalated nitriles (Scheme 1c).^[^ ^9^ ^]^ The coordination of the metalated entities is dictated by multiple factors, with the nature of the metal cation and its chemical environment playing a significant role.

Hence, solid‐state X‐ray diffraction (XRD) characterization provides a valuable picture of the variety of binding modes in metalated nitrile structures.^[^ ^9^, ^11^, ^12^ ^]^ Many of the reported structures suggest a strong preference for the formation of the N‐metalated species among alkali metals, as evidenced by N‐lithiated monomeric,^[^ ^13^ ^]^ homobimetallic complexes,^[^ ^14^, ^15^, ^16^ ^]^ polymers,^[^ ^17^ ^]^ and even a homotetrametallic N‐sodiated species.^[^ ^18^ ^]^ In contrast, Zn(II), Cu(II), and Ni(II) frequently furnish C‐metalation.^[^ ^19^, ^20^, ^21^, ^22^, ^23^, ^24^, ^25^, ^26^ ^]^ However, the metalation site is not determined solely by the nature of the metal. As an illustration, N‐metalation of electron‐deficient tricyanomethanide (C(CN)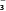) with Ni(II) has also been characterized by X‐ray crystallography.^[^ ^27^ ^]^

Though XRD reveals solid‐state preferences, it may not reflect solution behavior. Lithiated nitriles can exist as either solvent‐separated,^[^ ^28^ ^]^ mononuclear,^[^ ^29^, ^30^ ^]^ or polynuclear species,^[^ ^30^, ^31^ ^]^ illustrating their complex solution speciation. Their structure and reactivity depend on ligand denticity and sterics,^[^ ^32^, ^33^ ^]^ solvent,^[^ ^28^, ^30^, ^31^, ^34^ ^]^ substrate nature,^[^ ^35^ ^]^ and even temperature,^[^ ^34^ ^]^ making direct extrapolation from solid‐state data hazardous.

Computational chemistry can be a tool in unravelling solution structure.^[^ ^36^, ^37^, ^38^, ^39^, ^40^, ^41^, ^42^, ^43^, ^44^, ^45^, ^46^ ^]^ Additionally, it allows completing the structural characterization by analysis of electronic properties. In this context, the combination of geometry optimization and natural bond orbital (NBO) analysis was recently used to clarify the roles of alkali metal nature and solvent coordination in governing the bonding to biphenylene.^[^ ^47^ ^]^ We thus resort on a similar approach to unravel the factors that control the balance between C‐ and N‐metalation of acetonitrile by alkali metals and Cu(I) as a function of the nature of the metal coordination sphere.

## 2 Results and Discussion

### 2.1 Defining Geometrical and Electronic References

**Figure 1:**
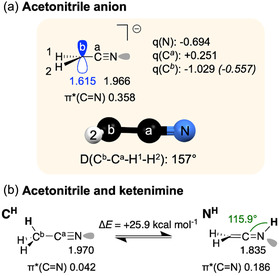
Electronic structures of a) acetonitrile anion and b) acetonitrile and ketenimine. Occupancies of the lone pairs and antibonding orbitals are given just below the structures. Atomic NPA charges (*q*, in *e*) are provided in plain text, with charges for CH_2_ groups shown in parentheses.

The longstanding debate surrounding charge delocalization in the acetonitrile anion [NCCH_2_]^−^ has been extensively documented in the literature.^[^ ^10^, ^48^, ^49^ ^]^ To establish a foundation for the forthcoming investigation of metalated acetonitrile, the geometry and electronic structure of [NCCH_2_]^−^ in the absence of a counter‐ion had to be recomputed at our computational level, using NBO calculations (**Figure** **1**a). This will serve as a reference point for assessing the influence of the cation nature on the electronic structure of [NCCH_2_]^−^. This anion exhibits the expected feature of a cyanide functional group, which is a short terminal C^*a*^≡N triple bond (*d*(C^*a*^—N): 1.191 Å, see **Table** **1**, entry 1) adjacent to a longer C^*a*^—C^*b*^ single bond (*d*(C^*a*^—C^*b*^ ): 1.379 Å). It also features two lone pairs: a fully occupied *sp* lone pair at the nitrogen atom and a partially occupied (1.615) *p* lone pair at the *α* position of the cyanide group (C^*b*^ atom). In line with previous computational and experimental studies,^[^ ^10^, ^48^, ^49^ ^]^ the negative NPA charge is primarily localized on the C^*b*^ atom (*q*(C^*b*^): –1.029 *e*). Structurally, this deprotonated form exhibits a small pyramidalization at the C^*b*^ center (dihedral *D*(C^*b*^—C^*a*^—H^1^—H^2^): 157°, see Figure 1a). This anion can serve as a model system for solvent‐separated ion pairs, in which the [NCCH_2_]^−^ anion and its counter‐ion coexist in independent solvation spheres (e.g., lithiated nitriles in highly coordinating solvents or solvent mixtures).^[^ ^28^ ^]^

**Table 1:** Impact of explicit solvent coordination on the structure of lithiated nitriles. Distances *d* are given in Å; angles *A* are given in °.

Entry	Y^MX^	*d*(C^*a*^—C^*b*^)	*d*(C^*a*^—N)	*A*(C^*a*^—N—M)	**C** ^MX^	*d*(C^*a*^—C^*b*^)	*d*(C^*a*^—N)	*A*(C^*a*^—C^*b*^—M)
1	**Acetonitrile anion**	1.379	1.191	–	–	–	–	–
2	**N** ^**H**^	1.313	1.229	116	**C** ^**H**^	1.451	1.162	110
3	**N** ^**Li**^	1.352	1.197	180	**C** ^**Li**^	1.409	1.179	105
4	**N** ^**Li(THF)1**^	1.354	1.196	180	**C** ^**Li(THF)1**^	1.406	1.189	102
5	**N** ^**Li(THF)2**^	1.357	1.195	179	**C** ^**Li(THF)2**^	1.401	1.182	94
6	**N** ^**Li(THF)3**^	1.361	1.194	153	**C** ^**Li(THF)3**^	1.396	1.184	100
7	**Bim** ^**NN**^	1.342	1.205	140	–	–	–	–
8	**Bim** ^**NN(THF)**^	1.346	1.201	143	–	–	–	–
9	**Bim** ^**NC**^	1.385	1.185	137	–	–	–	96
10	**Bim** ^**NC(THF)**^	1.384	1.184	142	–	–	–	98
11	**N** ^**Cs**^	1.367	1.195	175	**C** ^**Cs**^	1.193	4.008	57
12	**N** ^**K**^	1.362	1.196	179	**C** ^**K**^	1.193	3.094	64
13	**N** ^**Na**^	1.358	1.196	179	**C** ^**Na**^	1.403	1.181	103

To evaluate the impact of covalent bonding on the electronic structure of metalated nitriles, we next investigated the topology of two tautomeric protonated species (Figure 1b and Table 1, entry 2), featuring C^*b*^—H (nitrile form **C** ^**H**^) or N—H (ketenimine form **N** ^**H**^) bonds. Expectedly, acetonitrile **C** ^**H**^ is thermodynamically favored by an electronic energy difference (Δ*E*) of 25.9 kcal mol^−1^ relative to the ketenimine **N** ^**H**^. In the C‐protonated acetonitrile molecule **C** ^**H**^, the electronic structure remains very similar to [NCCH_2_]^−^, the lone pair at C^*b*^ now forming a covalent bond with H. In contrast, formation of the N—H bond is associated with a geometrical rearrangement (*d*(C^*a*^—C^*b*^): 1.313 Å, *d*(C^*a*^—N): 1.229 Å in Table 1, entry 2), with formation of two double bonds (C^*b*^=C^*a*^=N). Concerning the N lone pair, higher *p* character and smaller population are observed in the ketenimine tautomer **N** ^**H**^ (*sp* ^2^: N 39% *s*, 61% *p*, 1.835 *e*) than in the acetonitrile (*sp*: 54% *s*, 46% *p*, 1.970 *e*). These two clearly defined tautomer forms will serve as references for strongly localized structures.

### 2.2 Lithiated Species

**Figure 2:**
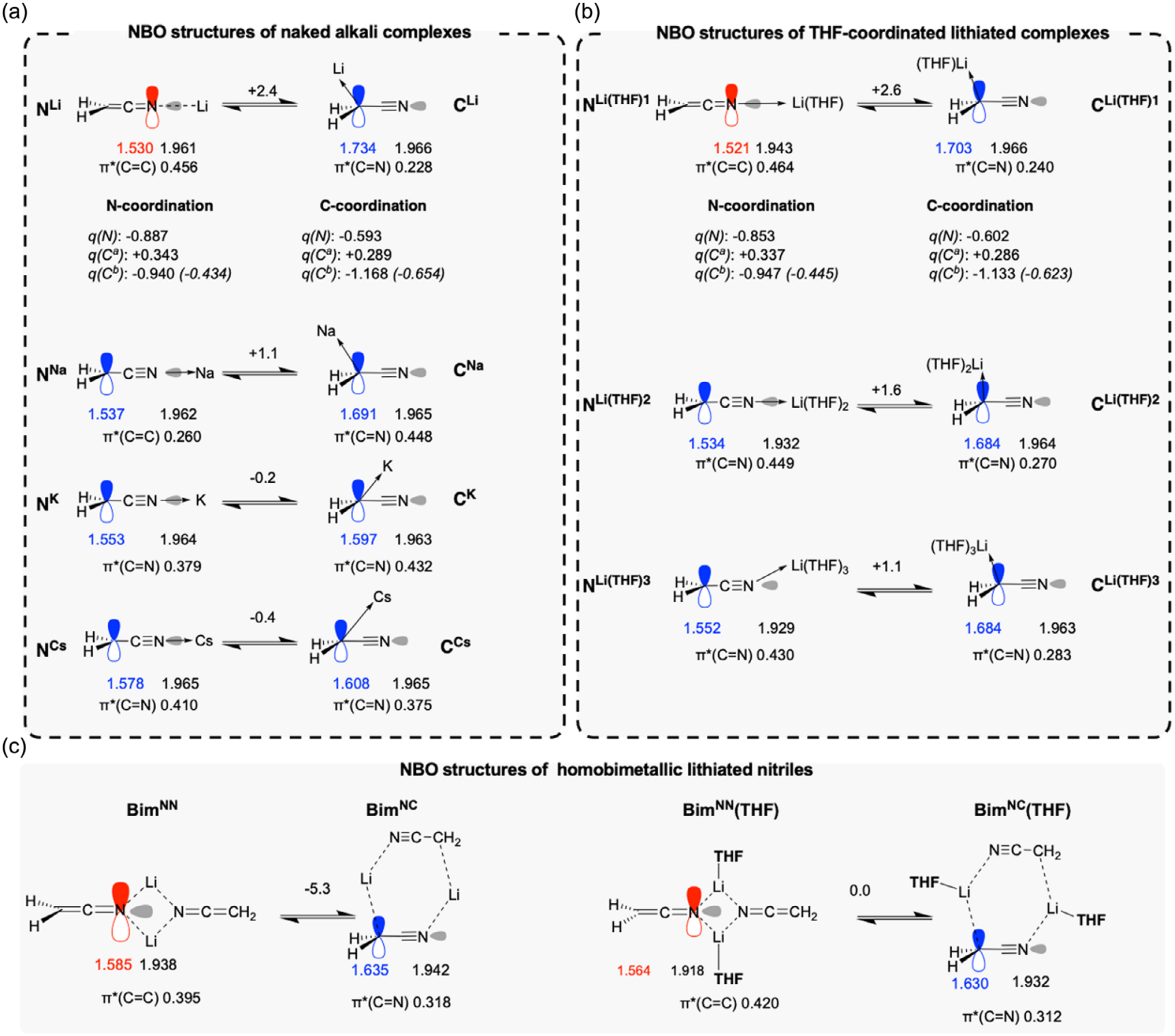
Electronic structures of a) monomeric alkali complexes, b) monomeric THF‐coordinated lithiated complexes, and c) homobimetallic lithiated complexes. Occupancies of the lone pairs and selected antibonding orbitals are given just below the structures. Atomic NPA charges (*q*, in *e*) are provided in plain text, with charges for CH_2_ groups shown in parentheses. The difference in electronic energy (Δ*E*, in kcal mol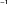) between N‐ and C‐metalation is given above the arrows, using N‐metalated complex as a reference.

We investigated how decreasing the covalency of the C—M or N—M bond would affect the bonding of [NCCH_2_]^−^. Numerous results from the literature suggest that lithium cation preferentially coordinates the nitrogen atom, to form monomeric or dimeric complexes.^[^ ^9^ ^]^ We first examined the coordination of a “naked” lithium cation with the N or C^*b*^ atoms (**Figure** **2**a). While such a species is unrealistic in solution, it will serve the discussion as an extreme case, in which cation polarization is unaffected by solvent coordination. Using a Li^+^, the N‐lithiated form (**N** ^**Li**^) is found to be slightly favored over the C‐lithiated one (**C** ^**Li**^) (Δ*E *= + 2.4 kcal mol^−1^). Notably, in **N** ^**Li**^, lithium interacts with the nitrogen atom along the C^*a*^—N axis (C^*a*^—N—Li: 180°, in Table 1, entry 3). The short C^*a*^—C^*b*^ distance (*d*(C^*a*^—C^*b*^): 1.352 Å) is closer to that observed in the protonated ketenimine **N** ^**H**^ (*d*(C^*a*^—C^*b*^) 1.313 Å, entry 2). The NBO analysis of **N** ^**Li**^ reveals that two lone pairs (*p*(N): 100% *p*, 1.530 *e*, and *sp*(N): 50 % *s*, 50 % *p*, 1.961 *e*) are located at the nitrogen atom, whereas both C^*b*^=C^*a*^ and C^*a*^=N are analyzed as double bonds. In contrast, C‐lithiation results in the formation of a C‐metalated nitrile **C** ^**Li**^, where the lone pairs are distributed as *sp*(N) (16 % *s*, 84% *p*, 1.734 *e*) and *p*(C^*b*^) (53% *s*, 47% *p*, 1.966 *e*), with a single C—C and a triple C≡N bond, similar to [NCCH_2_]^−^ (Figure 1 and 2a). This change in the location of the lone pairs is interesting, as it suggests that the lithium cation is polarizing enough to displace the *p*(C^*b*^) lone pair of [NCCH_2_]^−^ on the N atom in the N‐bonded structure.

Nevertheless, numerous studies from the literature have evidenced that organolithium compounds are highly sensitive to chemical environment effects—particularly solvent coordination.^[^ ^50^, ^51^, ^52^, ^53^, ^54^ ^]^ Therefore, we investigated the impact of THF coordination on the electronic structure of lithiated species (Figure 2b and Table 1, entry 4). Bond distances and NBO analyses of monosolvated N‐ and C‐lithiated complexes **N** ^**Li(THF)1**^ and **C** ^**Li(THF)1**^ reveal geometries and electronic structures nearly identical to their “naked” counterparts. Strikingly, coordinating a second THF to N‐lithiated ketenimine shifts the *p*(N) lone pair (100% *p*, 1.521 *e* in **N** ^**Li(THF)1**^) toward C^*b*^ atom (100% *p*, 1.534 *e* in **N** ^**Li(THF)2**^). This displacement is also associated with the formation of a single C—C and a triple C≡N bonds, consistent with the electronic structure of a nitrile group (Figure 2b). Despite these different electronic populations, **N** ^**Li(THF)1**^ and **N** ^**Li(THF)2**^ display quasi‐identical geometries (distances in Table 1 change within 0.003 Å, see entries 4 and 5). Notably, the short *d*(C—N) distance (1.357 Å) lies closer from the protonated ketenimine **N** ^**H**^ (1.313 Å) than from the protonated nitrile **C** ^**H**^ (1.451 Å) (Figure 1, entry 2), thus creating a discrepancy between the nitrile‐like electronic structure and the ketenimine‐like geometry in species **N** ^**Li(THF)2**^. The coordination of additional THF molecules has no more impact on the geometry and NBO structures, with the exception of the C^*a*^—N—Li bond angle that deviates from linearity, decreasing to 153° (see Table 1, entry 6 and cartesian coordinates in Supporting Information).

Energetically, the influence of solvent coordination on the N‐ versus C‐lithiation balance appears limited (Figure 2b). Though N‐lithiation remains systematically favored, this slight preference is gradually diminished by THF coordination (Δ*E* = −2.4, −2.6, −1.6 and −1.1 kcal mol^−1^ for the coordination of 0–3 THF).

The ability of lithiated species to form polymetallic aggregates has been evidenced by numerous studies.^[^ ^14^, ^15^, ^16^, ^17^, ^30^, ^31^ ^]^ The structure and electronic properties of N‐lithiated homobimetallic aggregates were also investigated (Figure 2c and Table 1, entries 7–10). Geometric data and NBO calculations (i.e., two double C=C and C=N bonds, as well as the presence of *sp*(N) and *p*(N) lone pairs, see Table 1 entry 7 and Figure 2c) indicate that the ketenimine pattern observed in **N** ^**Li**^ is retained in the dimeric form **Bim** ^**NN**^. An alternative dimeric structure **Bim** ^**NC**^ where each lithium coordinates both a C^*b*^ atom and a nitrogen atom can also be proposed. In this dimer, a cyanide functional group is suggested based on NBO analysis (see Figure 2), whereas geometrical data remain inconclusive. Energetically, this mixed N,C‐coordinated complex is favored over **Bim^**NN**^** by Δ*E* = −5.3 kcal mol^−1^. Coordination of solvent molecules to **Bim** ^**NN**^ and **Bim** ^**NC**^ has no effect on the structure (Table 1, entries 9 and 10) and bonding characteristics of the complexes (**Bim** ^**NN**^ **(THF)** and **Bim** ^**NC**^ **(THF)**), which become isoenergetic.

As a conclusion, the chemical environment surrounding the Li center (i.e., solvent coordination, aggregation state) plays a critical role in shaping the structure of the N‐lithiated complexes. When up to one solvent molecule is coordinated, as well as in homobimetallic complexes where Li^+^ coordinates exclusively to nitrogen atoms, the geometry and electronic structure resemble those of N‐lithiated ketenimines. In contrast, the coordination of more than two THF leads to the formation of a cyanide functional group despite the unaffected N‐coordination, which is also observed in homobimetallic complexes where Li^+^ interacts with both C^*b*^ and N atoms.

### 2.3 Comparison with Other Alkali Cations

In order to further elucidate how cation polarization modulates the balance between N‐ and C‐metalation, we examined the effects of larger alkali cations M^+^ (M = Na, K, Cs) on the electronic structure of [NCCH_2_]^−^. In the C‐metalated series, the N—C^*a*^—M angle varies with the nature of the cation (see **N** ^**M**^ complexes in Figure 2a, and entries 11–13 in Table 1). This variation reflects a change in coordination modes, shifting from an *η* ^1^ mode with Li^+^ and Na^+^ (105° and 103°) to a bridging mode with K^+^ and Cs^+^ (64° and 57°).

In contrast, N‐metalation (see **N** ^**M**^ complexes in Figure 2a) consistently occurs along the C^*a*^—N axis. However, NBO analyses reveal that these softer cations M^+^ are not polarizing enough to change the position of the lone pairs, and C≡N bonds are consequently retained whatever the metal bonding site. From a geometrical point of view, the bond lengths $d(C^{a}-C^{b})$ (1.367–1.358 Å, Table 1 entries 11–13) and $d(C^{a}-N)$ (1.195–1.196 Å) are nearly invariant across the series, and closely resemble those in the ketenimine complex **N** ^**Li**^ ($d(C^{a}-C^{b})$: 1.352 Å, $d(C^{a}-N)$: 1.197 Å, see Table 1 entry 3). A discrepancy in the attribution of nitrile/ketenimine thus appears between geometry and NBO structure. The NBO analyses provide herein a valuable complement to structural data, enabling discrimination between N‐metalated nitriles and ketenimines where geometry alone is inconclusive.

The equilibrium between N*‐* and C*‐*metalated species reflects the softening of the cation binding (Figure 2b). In line with a *η* ^1^‐localized C‐binding mode, Li^+^ and Na^+^ display a significant preference for this structure. In contrast, the equilibrium between the delocalized *η* ^3^‐binding and N‐only binding of Cs^+^ and K^+^ is quasi‐neutral.

From these results, it seems that Li^+^ is able to displace the lone pair from C^*b*^ to the N atom. The differences observed for the N—H and N—Li bonds both geometrically (C^*a*^—N—M angle value equal to 116° in **N** ^**H**^ and 180° in **N** ^**Li**^) and electronically^[^ ^55^ ^]^ show that a covalent bond between N and H is formed in the case of **N** ^**H**^ whereas none is obtained for **N** ^**Li**^. We thus propose that the lone pair localization at N in **N** ^**Li**^ is associated with the strong polarizing effect of the Li^+^ highly localized positive charge.

### 2.4 Analogy with an Electric Field

**Figure 3:**
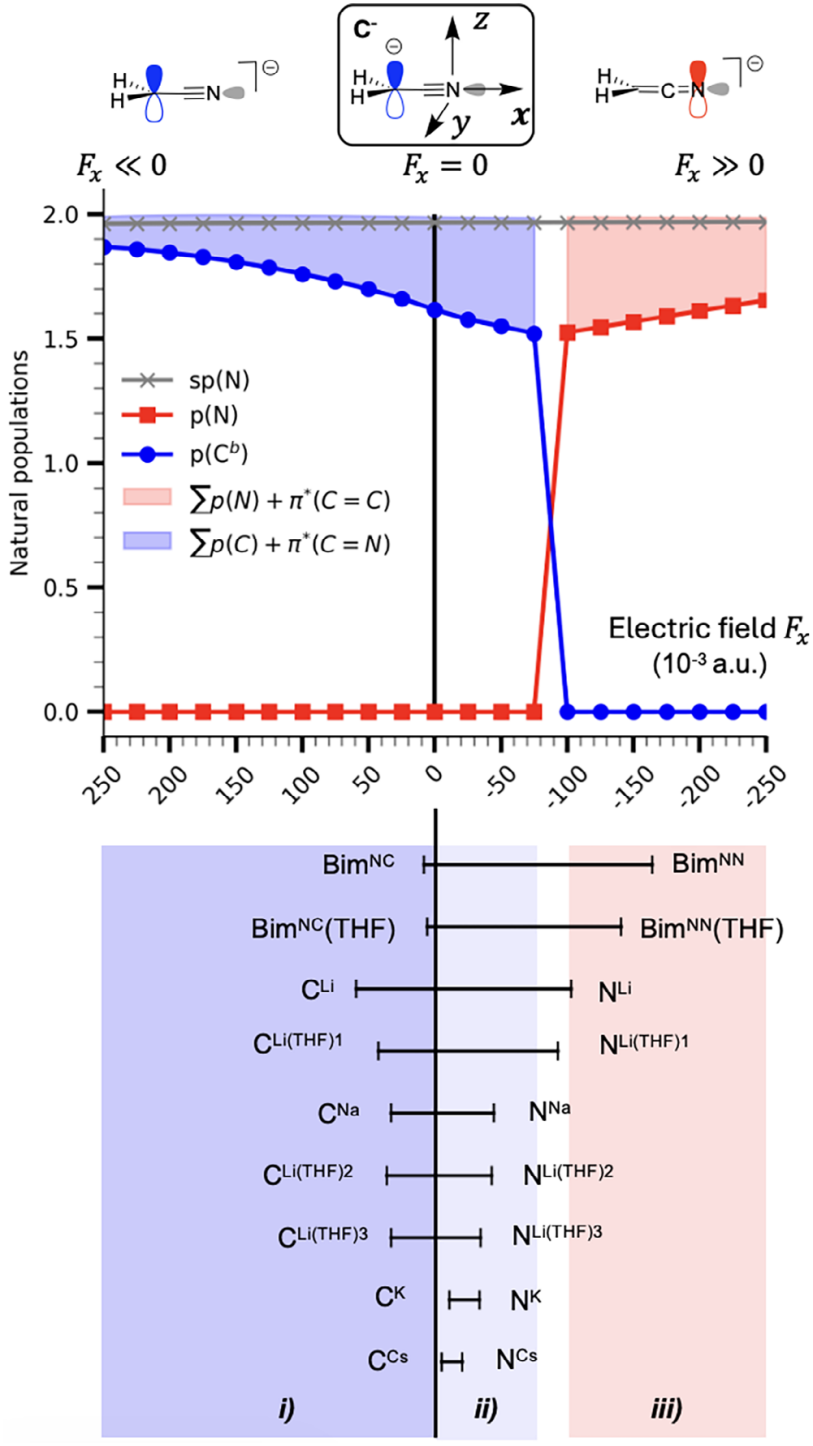
Impact of an electric field *F*(*x*) applied along the *x*‐axis on the populations of the lone pairs (upper part), and comparison with the populations obtained for mono‐ and bimetallic metalated nitriles (lower part). The straight black line (*F*_*x*_ = 0 a.u., *p*(C^*b*^): 1.615 *e*) corresponds to the acetonitrile anion in the absence of any electric field. Region (i) shows an increase in the *p*(C^*b*^) lone pair population, region (ii) a decrease, and region (iii) a progressive filling of the *p*(N) lone pair.

To further illustrate the critical impact of cation polarizing effect on the mesomeric balance in metalated species, we investigated the effect of an external electric field (*F*_*x*_) applied in the same direction as the one created by the charge, which is along the C^*a*^—N bond (*x* axis, **Figure** **3**) in [CH_2_CN]^−^. This computational experiment provides a convenient conceptual framework to probe the influence of a polarizing environment on resonance between nitrile and ketenimine forms. Applying a positive electric field slowly and continuously increases the occupancy of the *p*(C^*b*^) orbital (from 1.615 *e* with no field to 1.868 *e* with 0.250 a.u. field, the maximum applied) while reducing the population of the antibonding π*(C=N) orbital (from 0.373 *e* with no field, to 0.122 *e* for *F*_*x*_ = 0.250 a.u.). This effect is analogous to that observed when hard cations, such as lithium (*p*(C^*b*^): 1.734 *e* in **N** ^**Li**^) and sodium (*p*(C^*b*^): 1.691 *e* in **N** ^**Na**^), coordinate to the C^*b*^ atom of [CH_2_CN]^−^ (Figure 3, region i)). Interestingly, this observation also holds for all the THF‐coordinated C‐lithiated complexes that were studied. In contrast, applying a moderate negative electric field ($F_{x}>-0.075$ a.u.) along the C^*a*^—N bond initially depletes the lone pair *p*(C^*b*^) (down to 1.521 *e*), leading to progressive filling of the antibonding π*(C=N) orbital (0.465 *e*, for *F*_*x*_ = –0.075 a.u.). This behavior mirrors the effect of coordinating Na^+^ (1.537 *e*), K^+^ (1.553 *e*), or Cs^+^ (1.578 *e*) cations to the nitrogen atom (Figure 3, region ii)) A similar trend is also observed in di‐ and tri‐solvated N‐lithiated complexes—all of them featuring a cyanide functional group. For a more negative electric field ($F_{x}<-0.075$ a.u.), a shift toward the electronic structure of the ketenimine form occurs. The *p*(N) lone pair forms and continues to populate as the field strength increases in magnitude, consequently depleting the π*(C=C) orbital. This effect is similar to what is observed in the case of naked and mono‐solvated N‐lithiated complexes, which are identified as ketenimine‐like structures (Figure 3, region iii)). Overall, these results demonstrate that the application of an electric field can modulate the electronic structure of [CH_2_CN]^−^ in a similar manner to that of cation coordination to C^*b*^ or N. We propose that the strength of the electric field induced by the cations is the major factor in shaping the electronic structure of N‐metalated nitriles and ketenimines when binding alkali metals. The concept of controlling bond polarity and electronic structure by manipulating local electric fields is not limited to metalated species. Similar effects have been recently observed for imine bonds in donor–acceptor covalent organic frameworks, where adjusting the polarity of C=N bonds through chemical environment or applied fields was shown to control charge separation and enhance photocatalytic performance.^[^ ^56^ ^]^ The modulation of this effect when resorting on a nonalkali metal is next examined.

### 2.5 N‐ and C‐Metalated Complexes of Cu(I)

**Figure 4:**
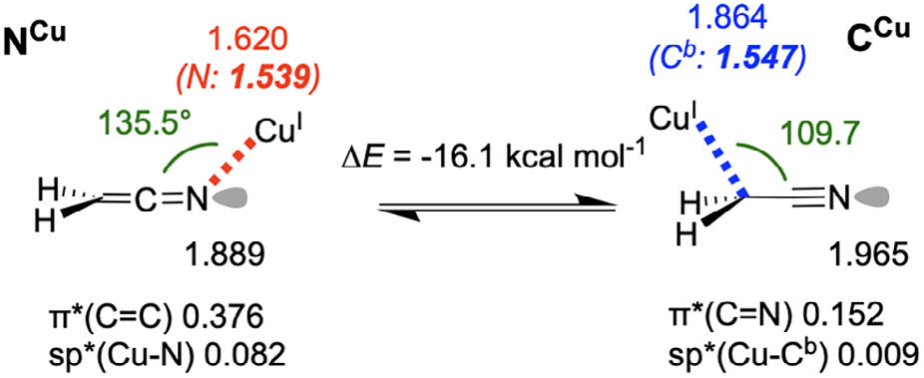
Electronic structures of ligand‐free copper complexes. Occupancies of the lone pairs and selected antibonding orbitals are given just below the structures.

**Figure 5:**
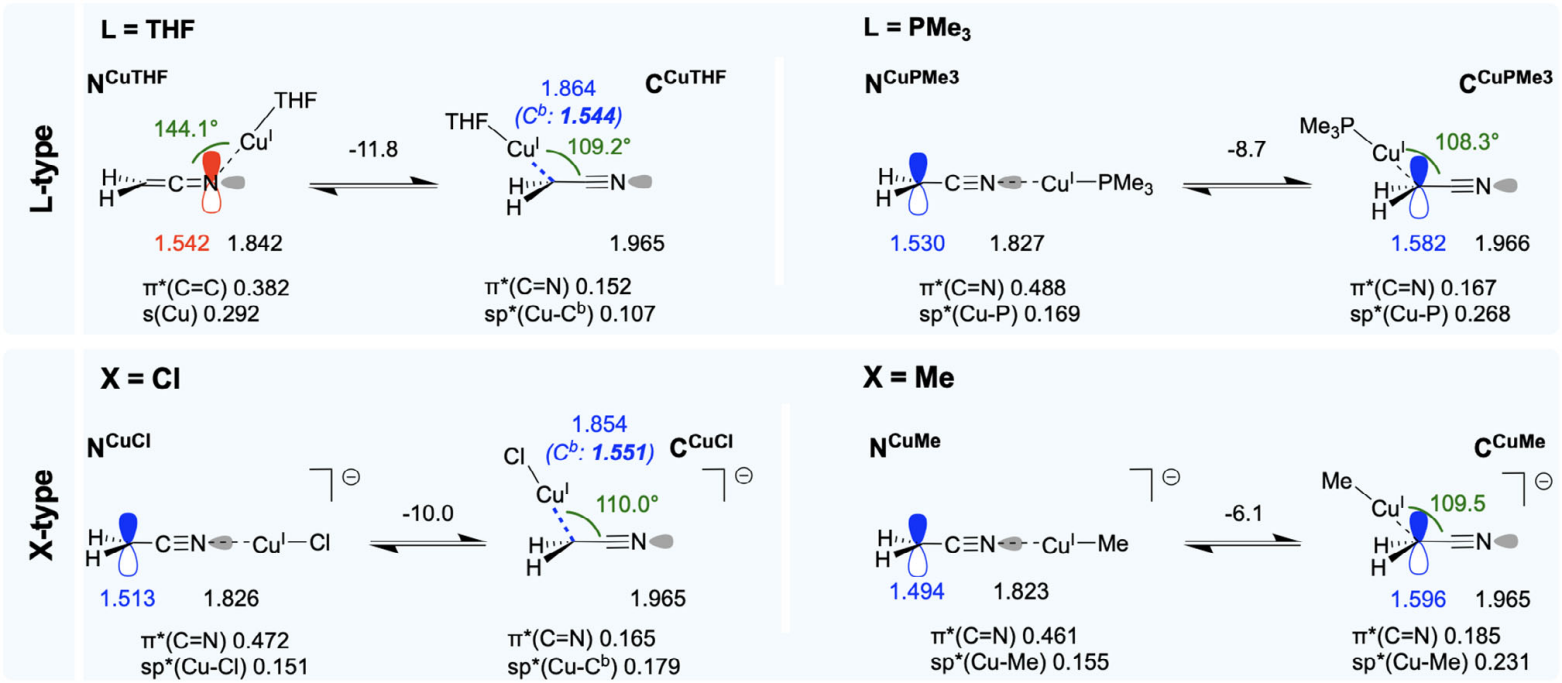
Electronic structures of LCu and XCu complexes. Occupancies of the lone pairs and selected antibonding orbitals are given just below the structures. Electronic energies (Δ*E*) are indicated above the arrows and are given in kcal mol^−1^.

Next, we chose to investigate the bonding characteristics of Cu(I) complexes (**Figure** **4** and **5**). With this 3*d* ^10^4*s* ^*0*^ cation, bonding primarily involves 4*s* orbitals (i.e., as for alkali metals) but exhibits significantly greater covalency. Using a single Cu^+^ cation as a ligand‐free model, two different binding modes could be obtained, the C‐metalated form being largely favored (Δ*E* = −16.1 kcal mol^−1^, relative to **N** ^**Cu**^, Figure 4). Coordination to the nitrogen atom (**N** ^**Cu**^) shows a C^*b*^—N—Cu value of 135.5°. A ketenimine‐like electronic structure is observed, with a *sp*(N) lone pair and a moderately populated Cu—N bond (1.620 electrons involved, 1.539 coming from N). Alternatively, coordination of the Cu^+^ cation with the C^*b*^ atom (**C** ^**Cu**^) yields the electronic structure of a C‐metalated nitrile, with a single *sp*(N) lone pair and a considerably more populated Cu—C^*b*^ bond (1.864 e) compared to the N‐metalated ketenimine **N** ^**Cu**^. Nevertheless, this simplified Cu(I) model does not take chemical environment effects into account, which will of course modulate the properties of the system.

Hence, we examined how the coordination of L‐ or X‐type ligands to the Cu(I) center shifts this equilibrium. The results obtained for all the stable metalated species are shown in Figure 5. Only the THF‐containing N‐metalated structure, which features the least *σ*‐donating L‐ligand, retains a ketenimine‐like electronic structure. The NBO analysis does not yield a covalent Cu—N bond but the population in the N lone pair (1.542 *e*) is nearly identical to the population in the Cu—N bond in **N** ^**Cu**^ (1.539 e). All other structures, either N‐ or C‐coordinated, exhibit a nitrile‐like electronic structure, with a triple C≡N bond and either a true lone pair at C or a Cu—C bond highly polarized at C. Most notably, Cu(PMe_3_) and Cu(Me) yield similar results despite a different global charge and thus a different electric field. Independently of the ligand nature, the equilibrium is shifted toward C‐metalation. Expectedly, the preference for C‐metalation can be diminished by coordinating strong *σ*‐donor ligands (L = PMe_3_, X = Me) in *trans*‐position to [CH_2_CN]^−^. These results illustrate that the chemical environment at the metal can be adjusted—through the choice of the solvent, ligands, and possibly additives—not only to decrease the preference for C‐coordination but also, and independently, to favor electronic structure with a lone pair at the terminal C.

## 3 Conclusion

**Scheme 2:**
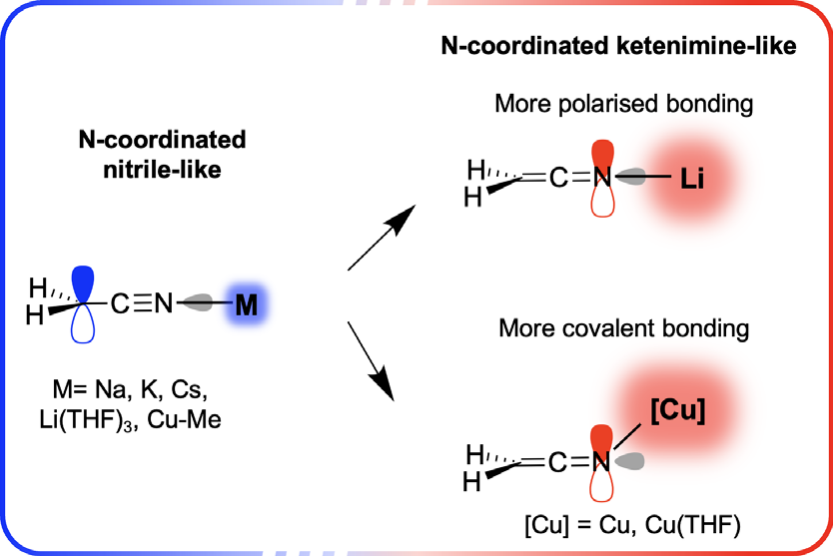
Shift of the electronic continuum from N‐metalated nitriles to N‐metalated kenetimines.

Computational studies have provided a clear illustration of the importance of chemical environment in modeling structures of metalated complexes. Consistently with previous reports,^[^ ^9^ ^]^ N‐lithiation is energetically preferred over C‐lithiation. For other alkali metals, a delocalized coordination to the whole π system is obtained with no significant site preference. However, in the fringes of the geometric continuum, a decorrelated electronic duality has emerged from NBO analyses of the N‐coordinated structures. In most cases, the electronic structure of the free anion [NCCH_2_]^−^ with localization of a lone pair at the terminal carbon is observed even for N‐coordinated species. However, a strongly polarizing cation (such as Li^+^) can induce transfer of the lone pair to the terminal N to form a ketenimine‐like electronic structure (see **Scheme** **2**). Hence, lithium exhibits a behavior that contrasts with other alkali metals—for which a cyanide functional group is systematically observed. In addition, solvent coordination on the N‐lithiated species induces a shift back from ketenimine to nitrile mesomeric forms, thus playing a crucial role in shaping its electronic structure. Formation of a covalent bond to N (such as N—H or N—Cu bonds) can also induce a ketenimine electronic structure in N‐bonded complexes (see Scheme 2), a shift strongly sensitive to the nature of the ligand at Cu. These covalently bonded structures are all associated with a preference for C‐coordination but it can be significantly tuned by the nature of the ligands, independently of the electronic structure observed.

## 4 Computational Details

All calculations were performed using the Gaussian 16 software (revision C.01).^[^ ^57^ ^]^ Geometries were optimized via frequency calculations at the B3PW91 level of theory,^[^ ^58^, ^59^ ^]^ incorporating Grimme D3 dispersion correction with Becke–Johnson damping (GD3BJ).^[^ ^60^ ^]^ A benchmark of computational methods is provided in Supporting Information. A continuum solvation model based on density (SMD) for THF was applied in all calculations.^[^ ^61^ ^]^ Explicit solvation is needed for properly modeling lithiated nitriles.^[^ ^62^ ^]^ Hence, for lithiated structures, additional calculations involving a mixed explicit–implicit representation of the solvent are carried out and reported. In this case, the THF molecules were preoptimized and then coordinated with the lithium one by one. The system was optimized again after each addition. No further conformational sampling was performed. For other alkali metals, the coordination of a THF molecule was found to be less exergonic and directional (see the Supporting Information for details), and only implicit solvation was used to discuss the impact of the nature of alkali metal on the balance between N‐ and C‐metalation.^[^ ^63^ ^]^ For the choice of basis sets, a split‐valence Pople basis set (6‐31++G**) was employed for light elements (C, H, N, O, Li, Na, P, Cl, and K),^[^ ^64^, ^65^, ^66^ ^]^ while fully relativistic Stuttgart–Dresden pseudopotentials with their associated basis sets were used for elements heavier than K (not included, i.e., Cu and Cs).^[^ ^67^, ^68^, ^69^ ^]^ Electronic structure analyses were conducted using the NBO7 package within Gaussian.^[^ ^70^ ^]^ All species examined in this study were generated through manual sampling, with particular attention given to exploring alternative coordination modes. Optimized geometries and associated energies are provided in an external file (structures.xyz). All the output files discussed in this article are available at https://doi.org/10.19061/iochem‐bd‐6‐582.

## Conflict of Interest

The authors declare no conflict of interest.

## Supporting information

Supplementary Material

## Data Availability

The data that support the findings of this study are available in the supplementary material of this article.

## References

[1] R. López , C. Palomo , Angew. Chem., Int. Ed. Engl. 2015, 54, 13170. 26387483 10.1002/anie.201502493

[2] R. López , C. Palomo , Angew. Chem. 2015, 127, 13366. 10.1002/anie.201502493 26387483

[3] R. Yazaki , N. Kumagai , M. Shibasaki , J. Am. Chem. Soc. 2009, 131, 3195. 19215140 10.1021/ja900001u

[4] R. Yazaki , N. Kumagai , M. Shibasaki , J. Am. Chem. Soc. 2010, 132, 5522. 20337453 10.1021/ja101687p

[5] A. Saito , N. Kumagai , M. Shibasaki , Tetrahedron Lett. 2014, 55, 3167.

[6] Y. Otsuka , H. Takada , S. Yasuda , N. Kumagai , M. Shibasaki , Chem. ‐ Asian J. 2013, 8, 354. 23208841 10.1002/asia.201201021

[7] Y. Yanagida , R. Yazaki , N. Kumagai , M. Shibasaki , Angew. Chem. 2011, 34, 8056. 10.1002/anie.201102467 21732508

[8] R. Qi , Q. Chen , L. Liu , Z. Ma , D. Pan , H. Wang , Z. Li , C. Wang , Z. Xu , Nat. Commun. 2023, 14, 3295. 37280209 10.1038/s41467-023-38871-1 PMC10244411

[9] X. Yang , F. F. Fleming , Acc. Chem. Res. 2017, 50, 2556. 28930437 10.1021/acs.accounts.7b00329

[10] J. P. Richard , G. Williams , J. Gao , J. Am. Chem. Soc. 1999, 121, 715.

[11] G. Boche , Angew. Chem., Int. Ed. Engl. 1989, 28, 277.

[12] G. Boche , Angew. Chem. 1989, 101, 286.

[13] I. Langlotz , M. Marsch , K. Harms , G. Boche , Z. Kristallogr. ‐ New Cryst. Struct. 1999, 214, 509.

[14] G. Boche , M. Marsch , K. Harms , Angew. Chem., Int. Ed. Engl. 1986, 25, 373.

[15] W. Zarges , M. Marsch , K. Harms , G. Boche , Angew. Chem., Int. Ed. Engl. 1989, 28, 1392.

[16] E. Iravani , B. Neumüller , Organometallics 2003, 22, 4129.

[17] G. Boche , K. Harms , M. Marsch , J. Am. Chem. Soc. 1988, 110, 6925.

[18] J. Barker , N. D. R. Barnett , D. Barr , W. Clegg , R. E. Mulvey , P. A. O’Neil , Angew. Chem., Int. Ed. Engl. 1993, 32, 1366.

[19] H. Brombacher , H. Vahrenkamp , Inorg. Chem. 2004, 43, 6054. 15360256 10.1021/ic049177a

[20] M. Krieger , R. O. Gould , K. Dehnicke , Z. Anorg. Allg. Chem. 2002, 628, 1289.

[21] Y. Yoshida , H. Ito , Y. Nakamura , M. Ishikawa , A. Otsuka , H. Hayama , M. Maesato , H. Yamochi , H. Kishida , G. Saito , Cryst. Growth Des. 2016, 16, 6613.

[22] T. A. Ateşin , T. Li , S. Lachaize , W. W. Brennessel , J. J. García , W. D. Jones , J. Am. Chem. Soc. 2007, 129, 7562. 17521188 10.1021/ja0707153

[23] A. M. Oertel , V. Ritleng , M. J. Chetcuti , L. F. Veiros , J. Am. Chem. Soc. 2010, 132, 13588. 20831173 10.1021/ja105368p

[24] J. Tehranchi , P. J. Donoghue , C. J. Cramer , W. B. Tolman , Eur. J. Inorg. Chem. 2013, 2013, 4077. 10.1002/ejic.201300328 PMC3885180 24415908

[25] X. Zhang , Z. Zhang , S. Xiang , Y. Zhu , C. Chen , D. Huang , Inorg. Chem. Front. 2019, 6, 1135.

[26] B. M. Schmidt , J. T. Engle , M. Zhang , I. Babahan , C. J. Ziegler , L. Jia , J. Organomet. Chem. 2016, 805, 94.

[27] S. Bruda , M. M. Turnbull , C. P. Landee , Q. Xu , Inorg. Chim. Acta 2006, 359, 298.

[28] P. R. Carlier , C. W.‐S. Lo , J. Am. Chem. Soc. 2000, 122, 12819.

[29] W. Bauer , D. Seebach , Helv. Chim. Acta 1984, 67, 1972.

[30] T. Strzalko , J. Seyden‐Penne , L. Wartski , J. Corset , M. Castella‐Ventura , F. Froment , J. Org. Chem. 1998, 63, 3287.

[31] D. Croisat , J. Seyden‐Penne , T. Strzalko , L. Wartski , J. Corset , F. Froment , J. Org. Chem. 1992, 57, 6435.

[32] T. Naota , A. Tannna , S.‐I. Murahashi , J. Am. Chem. Soc. 2000, 122, 2960. 10.1021/ja017868p 12059202

[33] D. A. Culkin , J. F. Hartwig , J. Am. Chem. Soc. 2002, 124, 9330. 12167001 10.1021/ja026584h

[34] R. Sott , J. Granander , G. Hilmersson , Chem. ‐ Eur. J. 2002, 8, 2081. 11981893 10.1002/1521-3765(20020503)8:9<2081::AID-CHEM2081>3.0.CO;2-Q

[35] M. Purzycki , W. Liu , G. Hilmersson , F. F. Fleming , Chem. Commun. 2013, 49, 4700. 10.1039/c3cc41703d 23589834

[36] L. M. Pratt , S. C. Nguyen , B. T. Thanh , J. Org. Chem. 2008, 73, 6086. 18646860 10.1021/jo800528y

[37] T. S. De Vries , A. Goswami , L. R. Liou , J. M. Gruver , E. Jayne , D. B. Collum , J. Am. Chem. Soc. 2009, 131, 13142. 19702308 10.1021/ja9047784 PMC2752606

[38] H. K. Khartabil , M. T. C. Martins‐Costa , P. C. Gros , Y. Fort , M. F. Ruiz‐López , J. Phys. Chem. B 2009, 113, 6459. 19344102 10.1021/jp809211y

[39] D. D. Dixon , M. A. Tius , L. M. Pratt , J. Org. Chem. 2009, 74, 5881. 20560562 10.1021/jo9008063

[40] L. M. Pratt , D. Jones , A. Sease , D. Busch , E. Faluade , S. C. Nguyen , B. T. Thanh , Int. J. Quantum Chem. 2009, 109, 34.

[41] N. Deora , P. R. Carlier , J. Org. Chem. 2010, 75, 1061. 20102232 10.1021/jo9016452

[42] B. Lecachey , H. Oulyadi , P. Lameiras , A. Harrison‐Marchand , H. Gérard , J. Maddaluno , J. Org. Chem. 2010, 75, 5976. 20677812 10.1021/jo101282m

[43] M. J. Houghton , D. B. Collum , J. Org. Chem. 2016, 81, 11057. 27749060 10.1021/acs.joc.6b02067 PMC5261255

[44] K. J. Jin , D. B. Collum , J. Am. Chem. Soc. 2015, 137, 14446. 26554898 10.1021/jacs.5b09524 PMC4762874

[45] E. H. Tallmadge , D. B. Collum , J. Am. Chem. Soc. 2015, 137, 13087. 26437278 10.1021/jacs.5b08207 PMC4765922

[46] S. O. Nilsson Lill , Computational Perspectives on Organolithiums, Wiley‐VCH, Weinheim, Germany 2014, pp. 33–52.

[47] A. M. Borys , A. E. F. Denjean , L. Vedani , D. Balcells , E. Hevia , Angew. Chem. Int. Ed. 2025, 64, e202501995. 10.1002/anie.202501995 39999419

[48] A. Abbotto , S. Bradamante , G. A. Pagani , J. Org. Chem. 1993, 58, 449.

[49] A. C. Hopkinson , M. H. Lien , K. Yates , P. G. Mezey , I. G. Csizmadia , J. Chem. Phys. 1977, 67, 517.

[50] A. de la Lande , C. Fressigné , H. Gérard , J. Maddaluno , O. Parisel , Chem. ‐ Eur. J. 2007, 13, 3459. 17225217 10.1002/chem.200601108

[51] A. Pierret , C. Lefebvre , P. C. Gros , C. Denhez , A. Vasseur , Eur. J. Org. Chem. 2023, 26, e202300954.

[52] H. Liang , A. M. Borys , E. Hevia , M.‐E. L. Perrin , P.‐A. Payard , J. Am. Chem. Soc. 2023, 145, 19989. 37646479 10.1021/jacs.3c06647

[53] M. de Giovanetti , S. H. Hopen Eliasson , A. C. Castro , O. Eisenstein , M. Cascella , J. Am. Chem. Soc. 2023, 145, 16305. 37471267 10.1021/jacs.3c04238 PMC10401704

[54] M. de Giovanetti , S. H. Hopen Eliasson , S. L. Bore , O. Eisenstein , M. Cascella , Chem. Sci. 2024, 15, 20355. 39574534 10.1039/d4sc04957h PMC11577267

[55] N(72%)‐H(28%) bonding natural orbital with *sp* ^2^ hybridization at N for N—H bond, and two 100% lone pairs at N either *sp* or only *p* hybridized for N‐Li.

[56] Z. Li , B. Cai , Q. Li , D. Zhang , Y. Liang , Y. Liu , Y. Jiao , A. Thomas , X. Zhao , Angew. Chem. Int. Ed. 2025, 64, e202509444. 10.1002/anie.202509444 40387783

[57] M. J. Frisch , G. W. Trucks , H. B. Schlegel , G. E. Scuseria , M. A. Robb , J. R. Cheeseman , G. Scalmani , V. Barone , G. A. Petersson , H. Nakatsuji , X. Li , M. Caricato , A. V. Marenich , J. Bloino , B. G. Janesko , R. Gomperts , B. Mennucci , H. P. Hratchian , J. V. Ortiz , A. F. Izmaylov , J. L. Sonnenberg , D. Williams‐Young , F. Ding , F. Lipparini , F. Egidi , J. Goings , B. Peng , A. Petrone , T. Henderson , D. Ranasinghe , et al., Gaussian 16, Revision C.01, Gaussian, Inc Wallingford CT 2019.

[58] A. D. Becke , Phys. Rev. A 1988, 38, 3098. 10.1103/physreva.38.3098 9900728

[59] J. P. Perdew , Y. Wang , Phys. Rev. B 1992, 45, 13244. 10.1103/physrevb.45.13244 10001404

[60] S. Grimme , S. Ehrlich , L. Goerigk , J. Comput. Chem. 2011, 32, 1456. 21370243 10.1002/jcc.21759

[61] A. V. Marenich , C. J. Cramer , D. G. Truhlar , J. Phys. Chem. B 2009, 113, 6378. 19366259 10.1021/jp810292n

[62] P. R. Carlier , J. D. Madura , J. Org. Chem. 2002, 67, 3832. 12027700 10.1021/jo0255633

[63] H. Gérard , R. Lucas‐Roper , R. Zerrouki , Carbohydr. Res. 2024, 535, 109012. 38157586 10.1016/j.carres.2023.109012

[64] W. J. Hehre , R. Ditchfield , J. A. Pople , The J. Chem. Phys. 1972, 56, 2257.

[65] P. C. Hariharan , J. A. Pople , Theor. Chim. Acta 1973, 28, 213.

[66] T. Clark , J. Chandrasekhar , G. W. Spitznagel , P. V. R. Schleyer , J. Comput. Chem. 1983, 4, 294.

[67] M. Dolg , U. Wedig , H. Stoll , H. Preuss , J. Chem. Phys. 1987, 86, 866.

[68] J. M. L. Martin , A. Sundermann , J. Chem. Phys. 2001, 114, 3408.

[69] D. Figgen , G. Rauhut , M. Dolg , H. Stoll , Chem. Phys. 2005, 311, 227.

[70] E. D. Glendening , J. K. Badenhoop , A. E. Reed , J. E. Carpenter , J. A. Bohmann , C. M. Morales , P. Karafiloglou , C. R. Landis , F. Weinhold , NBO 7.0, Theoretical Chemistry Institute, University of Wisconsin, Madison 2018.

