# Supplementary material for: Computational Experiments Probing the Adaptability of the [NCCH2]− Electronic Structure to Various Bonding Environments
Source: Chemphyschem. 2026 Jan 25;27(2):e202500580. doi: 10.1002/cphc.202500580 (PMC12833475; doi:10.1002/cphc.202500580)
Supplement: Supplementary file 1 — Supplementary Material [file CPHC-27-e202500580-s001.pdf]

Supporting Information

Chemical Environment Effects Dictate the Bonding Continuum in Metalated Nitriles

Jordan Rio*[a], Jean-François Brière[b], and Hélène Gérard[a]

[a]Laboratoire de Chimie Théorique (LCT), Sorbonne Université, CNRS, 4 Place Jussieu, 75005 Paris, France. Corresponding author: jordan.rio@sorbonne-universite.fr
[b]CNRS, INSA Rouen Normandie, Université Rouen Normandie, Université Caen Normandie, ENSICAEN, Institut CARMeN UMR 6064, 76000 Rouen, France

---

Table of Contents

1 Method Validation **S2**
1.1 Method validation on lithiated nitriles. S3
1.2 Method validation on transition-metal complexes. S5

2 THF-coordination to Na-, K- and Cs-complexes **S6**

3 Free-energy Calculations **S8**

References **S10**

1 Method Validation

We investigated the effects of the base and the method on lithiated nitriles, as well as some Neutrally and negatively charged copper-complexes. Electronic energy differences relative to *N*-metalated species (ΔE) are shown in **Figures S1**, **Figures S2**, **Figures S3** and **Figures S4**).

All the calculations were performed as single-point energies on the geometries optimised at the B3Pw91/6-31++G**/SMD(THF) level. A SDD pseudopotential with its associated triple ζ basis for Cu.[1, 2, 3] The SMD continuum model (THF) was added to all calculations,[4] except with calculations at a CCSD(T) level. The GD3BJ empirical dispersion correction was also included in every DFT calculations.[5] While the gaussian 16 (revision C01) software was used for DFT and MP2 calculations,[6] we used the orca code (version 5.0) for coupled-cluster calculations.[7]

An insight of the impact of the level of theory on the optimised geometries of lithiated nitriles is also provided in Tables S1 and S2.

1.1 Method validation on lithiated nitriles.

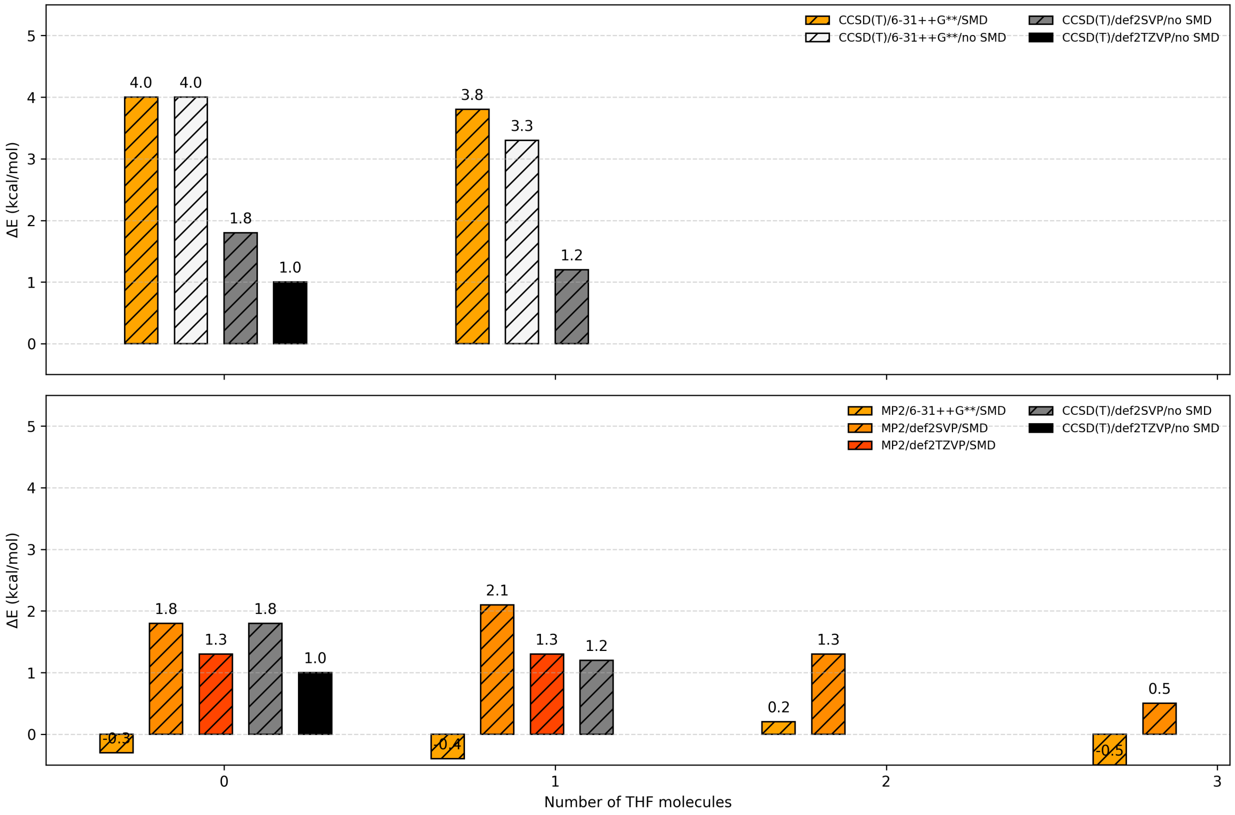

Figure S1. Impact of the basis set definition on the CCSD(T) and MP2 method, using N-lithiated species as a reference.

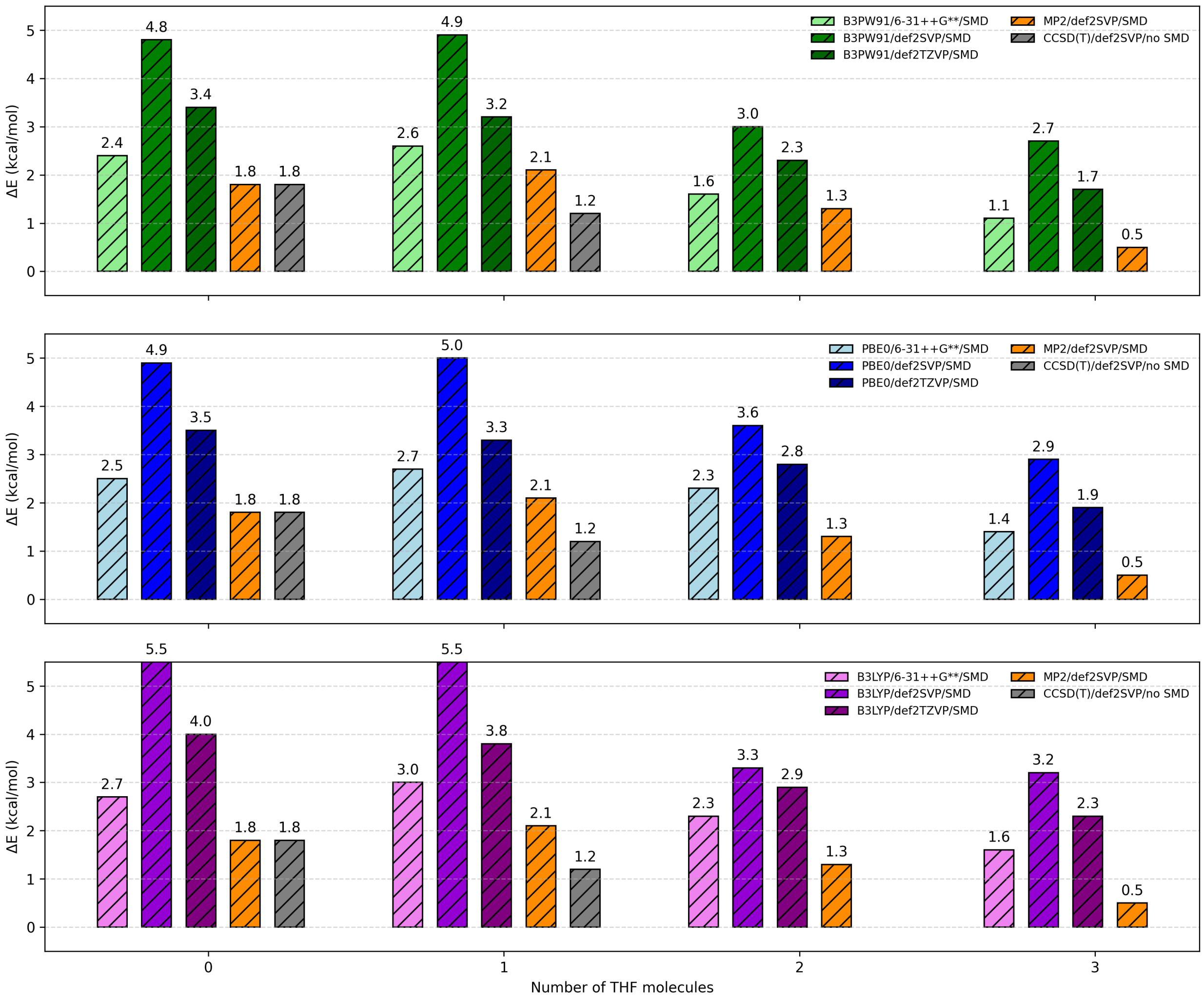

Figure S2. Comparison of electronic energies computed by DFT methods with CCSD(T) and MP2 methods, using N-lithiated species as a reference.

Table S1. Impact of basis set and functional on the structure of $\mathbf{N^{Li}}$ and $\mathbf{C^{Li}}$. Distances d are given in Å, angles A are given in °.

	$\mathbf{N^{Li}}$:	C^a-C^b	C^a-N	C^a-N-M	$\mathbf{C^{Li}}$:	C^a-C^b	C^a-N	C^a-C^b-M
B3PW91 / 6-31++G**		1.35	1.20	180		1.41	1.18	105
B3PW91 / def2SVP		1.35	1.20	179		1.42	1.17	106
B3PW91 / def2TZVP		1.35	1.19	180		1.40	1.17	104
PBE0 / 6-31++G**		1.35	1.20	179		1.41	1.18	106
PBE0 / def2SVP		1.35	1.20	180		1.42	1.17	107
PBE0 / def2TZVP		1.35	1.18	179		1.40	1.17	105
B3LYP / 6-31++G**		1.35	1.20	179		1.41	1.18	106
B3LYP / def2SVP		1.35	1.20	180		1.42	1.17	107
B3LYP / def2TZVP		1.35	1.19	180		1.41	1.17	105

Table S2. Impact of basis set and functional on the structure of $\mathbf{N^{Li(THF)1}}$ and $\mathbf{C^{Li(THF)1}}$. Distances d are given in Å, angles A are given in °.

	$\mathbf{N^{Li(THF)1}}$	C^a-C^b	C^a-N	C^a-N-M	$\mathbf{C^{Li(THF)1}}$	C^a-C^b	C^a-N	C^a-C^b-M
B3PW91 / 6-31++G**		1.36	1.19	153		1.40	1.18	100
B3PW91 / def2TZVP		1.36	1.18	152		1.39	1.17	99
PBE0 / def2TZVP		1.35	1.18	177		1.39	1.17	100

1.2 Method validation on transition-metal complexes.

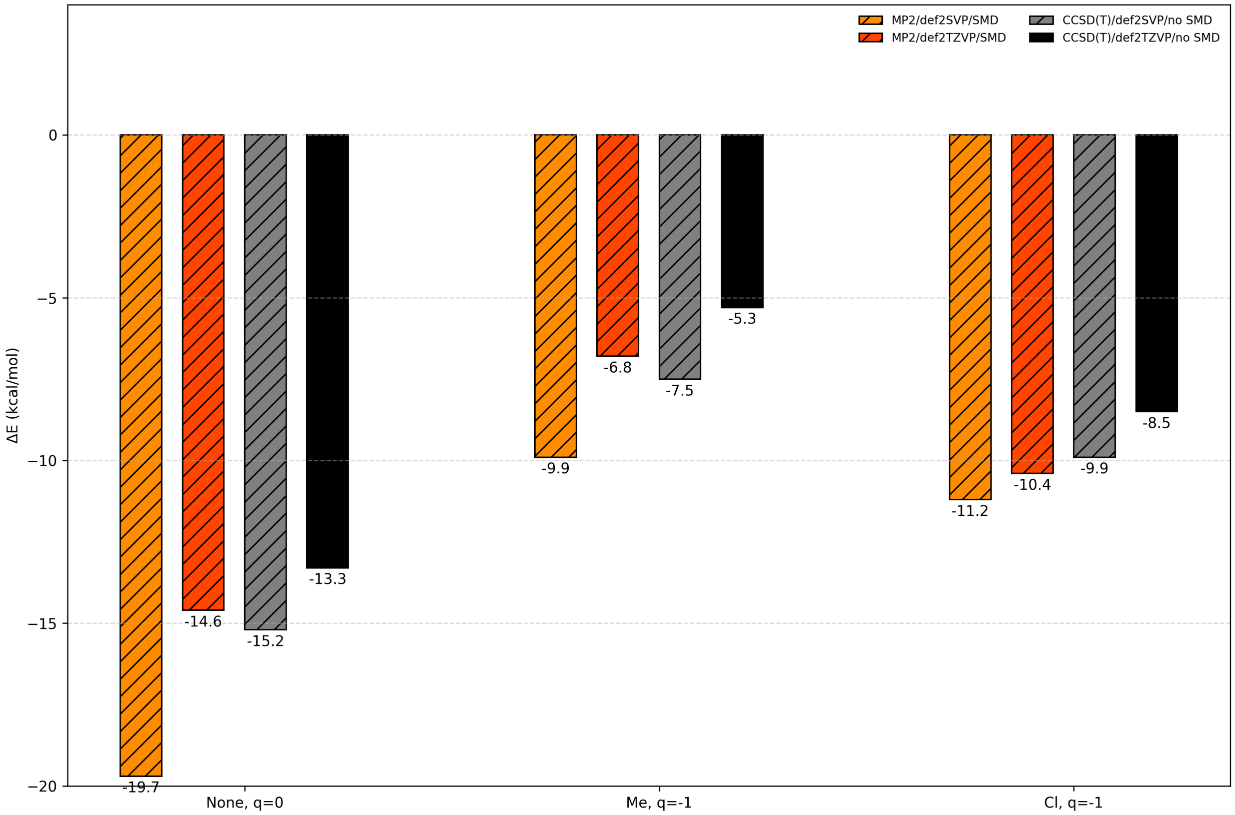

Figure S3. Impact of the basis set definition on the CCSD(T) and MP2 method with $[CH_2CN][CuX]^q$ (X= None, Me or Cl, $q = 0$ or -1), using N-metalated species as a reference.

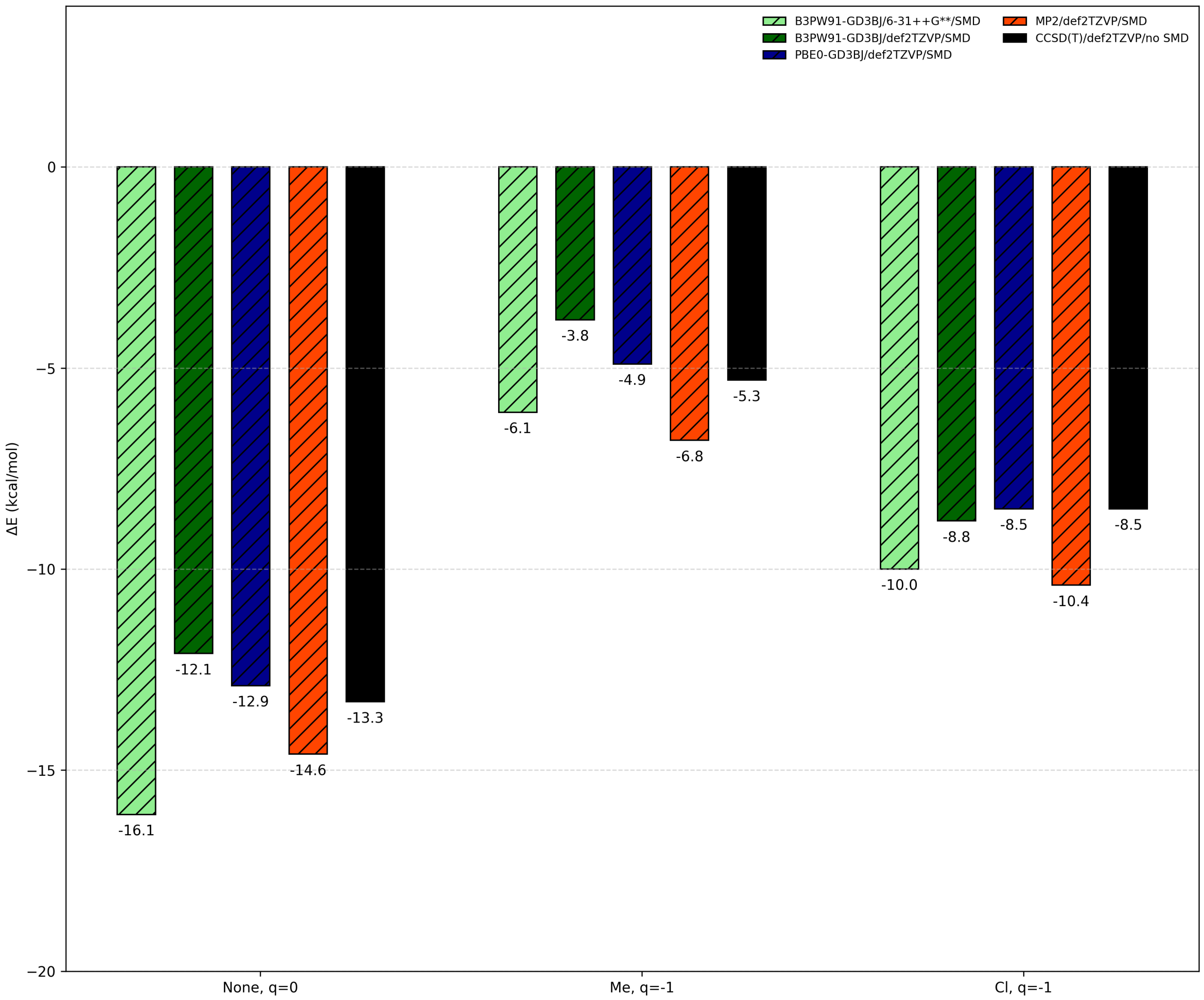

Figure S4. Comparison of electronic energies computed by DFT methods with CCSD(T) and MP2 methods, using N-metalated species as a reference.

2 THF-coordination to Na-, K- and Cs-complexes

The coordination of THF molecules to Na-, K- and Cs-complexes of C- and C-bound nitriles, using the level of theory used in the main study (B3PW91+GD3BJ/6-31++G**(C,H,N,O,Na, K)/SDD(Cs) and associated basis set/SMD(THF).

System	ΔG (ΔE)
Li$-$NCCH$_2$ + THF $\rightarrow$ Li$-$NCCH$_2$(THF)$_1$	**-9.8** *(-19.5)*
Li$-$CH$_2$CN + THF $\rightarrow$ Li$-$CH$_2$CN(THF)$_1$	**-9.1** *(-19.3)*
Na$-$NCCH$_2$ + THF $\rightarrow$ Na$-$NCCH$_2$(THF)$_1$	**-5.5** *(-15.1)*
Na$-$CH$_2$CN + THF $\rightarrow$ Na$-$CH$_2$CN(THF)$_1$	**-5.9** *(-15.1)*
K$-$NCCH$_2$ + THF $\rightarrow$ K$-$NCCH$_2$(THF)$_1$	**-2.1** *(-11.1)*
K$-$CH$_2$CN + THF $\rightarrow$ Na$-$CH$_2$CN(THF)$_1$	**-1.8** *(-11.3)*
Cs$-$NCCH$_2$ + THF $\rightarrow$ Cs$-$NCCH$_2$(THF)$_1$	**+4.9** *(-4.4)*
Cs$-$CH$_2$CN + THF $\rightarrow$ Cs$-$CH$_2$CN(THF)$_1$	**+4.5** *(-4.5)*

Table S3. Free-energies and electronic energies (Δ**G**(ΔE), in kcal mol^{-1}) obtained for the coordination of a single THF molecule N- and C-metalated nitriles (M= Li, Na, K, Cs).

System	ΔG (ΔE)
Li$-$NCCH$_2$ + 2 THF $\rightarrow$ Li$-$NCCH$_2$(THF)$_2$	**-14.8** *(-35.9)*
Li$-$CH$_2$CN + 2 THF $\rightarrow$ Li$-$CH$_2$CN(THF)$_2$	**-13.6** *(-36.6)*
Na$-$NCCH$_2$ + 2 THF $\rightarrow$ Na$-$NCCH$_2$(THF)$_2$	**-7.0** *(-28.2)*
Na$-$CH$_2$CN + 2 THF $\rightarrow$ Na$-$CH$_2$CN(THF)$_2$	**-7.3** *(-29.4)*

Table S4. Free-energies and electronic energies (Δ**G**(ΔE), in kcal mol^{-1}) obtained for the coordination of a second THF molecule to N- and C-metalated nitriles (M= Li, Na).

System	n=0	n=1
Li$-$NCCH$_2$(THF)$_n$ $\rightarrow$ Li$-$CH$_2$CN(THF)$_n$	2.4	2.6
Na$-$NCCH$_2$(THF)$_n$ $\rightarrow$ Na$-$CH$_2$CN(THF)$_n$	1.1	1.1
K$-$NCCH$_2$(THF)$_n$ $\rightarrow$ K$-$CH$_2$CN(THF)$_n$	-0.2	-0.4
Cs$-$NCCH$_2$(THF)$_n$ $\rightarrow$ Cs$-$CH$_2$CN(THF)$_n$	-0.4	-0.6

Table S5. Electronic energies (ΔE, in kcal mol^{-1}) obtained for the equilibrium of N- and C-metalated nitriles (M= Na, K, Cs) for different solvation states.

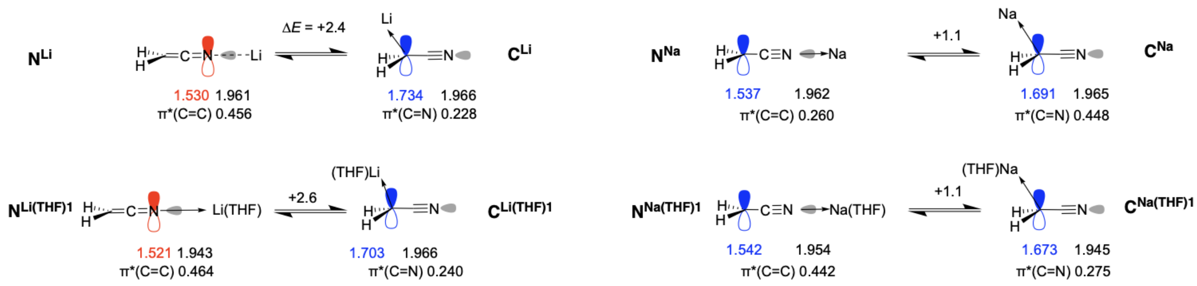

Figure S5. NBO structures of "naked" and THF-coordinated lithiated and sodiated nitriles. Electronic energies (ΔE) in kcal mol^{-1}.

3 Free-energy Calculations

We investigated the free energies of mono- and dimeric lithiated nitriles under different correction schemes, labeled [a–e]:

- No correction [a].
- SSC(1) [b]: Standard state correction (SSC: 1 mol/L for all species) combined without frequency scaling.
- SSC(1 + **THF**) [c]: Standard state corrections (SSC: 1 mol/L for all molecules except THF, which is 12.5 mol/L) without frequency scaling.
- SSC(1+THF) + **free space (toluene)** [d]: Standard state corrections (SSC: 1 mol/L for all molecules, except THF at 12.5 mol/L), without frequency scaling, and including free-space correction for toluene (0.133 mol/L, corresponding to toluene). Toluene was chosen as it is already implemented in the GoodVibes package, and possess a density (0.866) that is very similar to that of THF (0.867, compared to 0.886 for THF).
- SSC(1+THF) + **free space (H_2O)** [e]: Standard state corrections (SSC: 1 mol/L for all molecules, except THF at 12.5 mol/L), without frequency scaling, and including free-space correction for H_2O (0.008 mol/L). Water was chosen as it is already implemented in the GoodVibes package, and possess a density (1.000) that is very different to the density of THF (0.867, compared to 0.886 for THF), and a free volume that largely differs from toluene.

All free-energy values were obtained using the GoodVibes package,[8] and are reported in Table S6.

Complex	ΔE	ΔG (no free-space)			ΔG (with free-space)	
		no corr [a]	SSC(1) [b]	SSC(1+THF) [c]	SSC(1+THF) +FS(tol) [d]	SSC(1+THF) +FS(H_2O) [e]
LiC	0.0	0.0	0.0	0.0	0.0	0.0
LiN	-2.4	-3.0	-2.9	-2.9	-2.9	-2.9
LiC(THF)$_1$	-19.3	-8.8	-9.1	-10.6	-14.5	-16.2
LiN(THF)$_1$	-19.5	-9.3	-9.8	-11.3	-15.2	-16.9
LiC(THF)$_2$	-36.6	-12.1	-13.6	-16.6	-24.4	-27.7
LiN(THF)$_2$	-38.3	-17.0	-17.7	-20.7	-28.5	-31.8
LiC(THF)$_3$	-50.7	-12.9	-15.7	-20.1	-31.9	-36.8
LiN(THF)$_3$	-51.8	-16.4	-18.3	**-22.8**	**-34.5**	**-39.5**
LiC(THF)$_4$	-53.9	-2.7	-6.9	-12.9	-28.6	-35.1
LiN(THF)$_4$	**-56.9**	-6.5	-10.8	-16.7	-32.4	-38.9
μ(N,C)-[LiNCCH$_2$]$_2$	-20.7	-14.2	-14.9	-14.9	-16.9	-17.7
μ(N,C)-[LiNCCH$_2$(THF)$_1$]$_2$	-36.1	-18.6	-19.2	-20.6	-26.5	-29.0
μ(N)-[LiNCCH$_2$]$_2$	-18.0	-12.2	-12.7	-12.7	-14.7	-15.5
μ(N)-[LiNCCH$_2$(THF)$_2$]$_2$	-36.1	**-19.6**	**-19.9**	-21.4	-27.3	-29.8

Table S6. Relative corrected and uncorrected energies (ΔE, ΔG, in kcal mol^{-1}) of Lithiated complexes, using LiC as a reference. For dimeric species, the dimerisation reaction is defined as: LiC + n THF $\rightarrow \frac{1}{2}$ [LiX(THF)$_n$]$_2$. For each set of corrections, lowest free energies minima are in bold.

For a more intuitive comparison, the results are also visualized in Figure S6.

In the absence of corrections, N-metalation is favoured over C-metalation. Based on differences in electronic energies, the most stable species is LiN(THF)$_4$. However, according to the computed free-energies, the formation of LiN(THF)$_2$ is thermodynamically favoured (ΔG

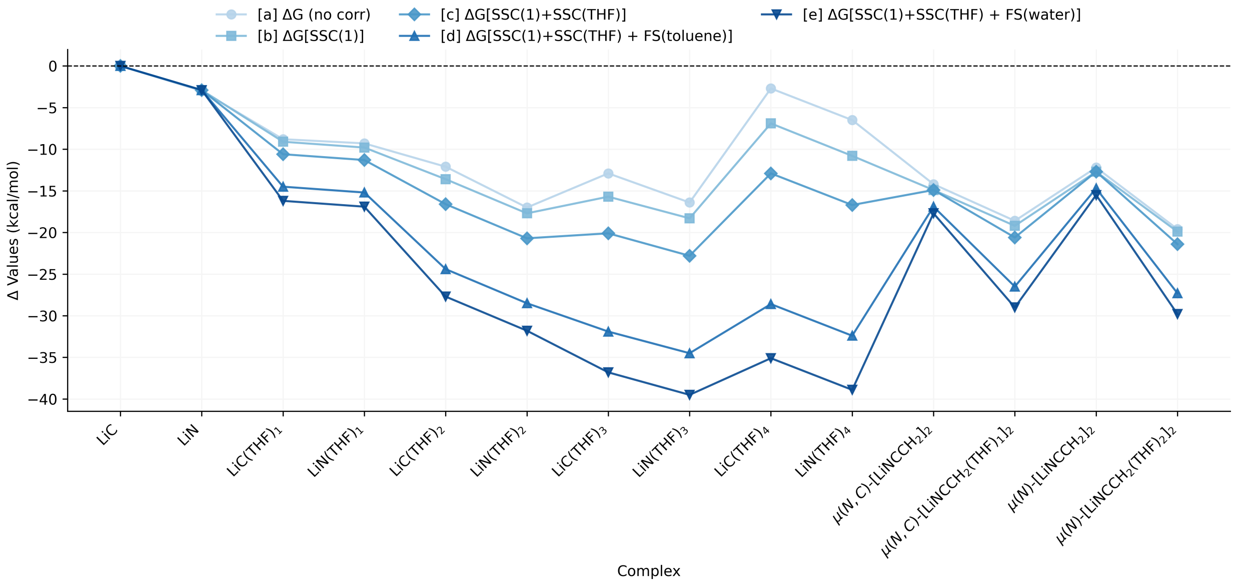

Figure S6. Corrected and uncorrected free-energies of mono- and di-meric lithiated complexes. For dimeric species, we defined the dimerisation as: LiC + n THF $\rightarrow \frac{1}{2}$ [LiX(THF)$_n$]$_2$ (n = 0 or 2). tol = toluene

= -17.0 kcal mol^{-}1) and competes with head-to-head (μ(N,N)-[LiNCCH$_2$(THF)$_1$]$_2$, ΔG = -19.6 kcal mol^{-}1) and head-to-tail (μ(N,C)-[LiNCCH$_2$(THF)$_1$]$_2$, ΔG = -18.6 kcal mol^{-}1)types solvated dimers.

Correcting the free energies shows that N-metalation remains favoured over C-metalation. However, the computed thermodynamically favoured species is the triply solvated N-lithiated species NLi(THF)$_3$ (ΔG = -19.6 kcal mol^{-1}).

References

[1] M. Dolg, U. Wedig, H. Stoll, and H. Preuss. Energy-adjusted ab initio pseudopotentials for the first row transition elements. *J. Chem. Phys.*, 86(2):866–872, January 1987. ISSN 1089-7690. doi: 10.1063/1.452288. URL http://dx.doi.org/10.1063/1.452288.

[2] Jan M. L. Martin and Andreas Sundermann. Correlation consistent valence basis sets for use with the stuttgart–dresden–bonn relativistic effective core potentials: The atoms ga–kr and in–xe. *J. Chem. Phys.*, 114(8):3408–3420, February 2001. ISSN 1089-7690. doi: 10.1063/1.1337864. URL http://dx.doi.org/10.1063/1.1337864.

[3] Detlev Figgen, Guntram Rauhut, Michael Dolg, and Hermann Stoll. Energy-consistent pseudopotentials for group 11 and 12 atoms: adjustment to multi-configuration dirac–hartree–fock data. *Chem. Phys.*, 311(1–2):227–244, April 2005. ISSN 0301-0104. doi: 10.1016/j.chemphys.2004.10.005. URL http://dx.doi.org/10.1016/j.chemphys.2004.10.005.

[4] Aleksandr V. Marenich, Christopher J. Cramer, and Donald G. Truhlar. Universal solvation model based on solute electron density and on a continuum model of the solvent defined by the bulk dielectric constant and atomic surface tensions. *J. Phys. Chem. B*, 113(18):6378–6396, April 2009. ISSN 1520-5207. doi: 10.1021/jp810292n. URL http://dx.doi.org/10.1021/jp810292n.

[5] Stefan Grimme, Stephan Ehrlich, and Lars Goerigk. Effect of the damping function in dispersion corrected density functional theory. *J. Comput. Chem.*, 32(7):1456–1465, March 2011. ISSN 1096-987X. doi: 10.1002/jcc.21759. URL http://dx.doi.org/10.1002/jcc.21759.

[6] M. J. Frisch, G. W. Trucks, H. B. Schlegel, G. E. Scuseria, M. A. Robb, J. R. Cheeseman, G. Scalmani, V. Barone, G. A. Petersson, H. Nakatsuji, X. Li, M. Caricato, A. V. Marenich, J. Bloino, B. G. Janesko, R. Gomperts, B. Mennucci, H. P. Hratchian, J. V. Ortiz, A. F. Izmaylov, J. L. Sonnenberg, D. Williams-Young, F. Ding, F. Lipparini, F. Egidi, J. Goings, B. Peng, A. Petrone, T. Henderson, D. Ranasinghe, V. G. Zakrzewski, J. Gao, N. Rega, G. Zheng, W. Liang, M. Hada, M. Ehara, K. Toyota, R. Fukuda, J. Hasegawa, M. Ishida, T. Nakajima, Y. Honda, O. Kitao, H. Nakai, T. Vreven, K. Throssell, J. A. Montgomery, Jr., J. E. Peralta, F. Ogliaro, M. J. Bearpark, J. J. Heyd, E. N. Brothers, K. N. Kudin, V. N. Staroverov, T. A. Keith, R. Kobayashi, J. Normand, K. Raghavachari, A. P. Rendell, J. C. Burant, S. S. Iyengar, J. Tomasi, M. Cossi, J. M. Millam, M. Klene, C. Adamo, R. Cammi, J. W. Ochterski, R. L. Martin, K. Morokuma, O. Farkas, J. B. Foresman, and D. J. Fox. *Gaussian 16 Revision C.01*, 2016. Gaussian Inc. Wallingford CT.

[7] Frank Neese. Software update: The orca program system—version 5.0. *WIREs Computational Molecular Science*, 12(5), March 2022. ISSN 1759-0884. doi: 10.1002/wcms.1606. URL http://dx.doi.org/10.1002/wcms.1606.

[8] Guilian Luchini, Juan V. Alegre-Requena, Ignacio Funes-Ardoiz, and Robert S. Paton. Goodvibes: automated thermochemistry for heterogeneous computational chemistry

data. *F1000Research*, 9:291, April 2020. ISSN 2046-1402. doi: 10.12688/f1000research.22758.1. URL http://dx.doi.org/10.12688/f1000research.22758.1.
